# Cervical Subcutaneous Emphysema and Pneumomediastinum Following Revision Septorhinoplasty With Autologous Costal Cartilage Graft: A Case Report

**DOI:** 10.7759/cureus.75709

**Published:** 2024-12-14

**Authors:** Özgür Kemal, Emre Demirel, Mehmet Çelebi

**Affiliations:** 1 Otolaryngology - Head and Neck Surgery, Ondokuz Mayıs University Faculty of Medicine, Samsun, TUR

**Keywords:** costal cartilage, post op pneumomediastinum, revision rhinoplasty, rhinoplasty surgery, subcutaneous emphysema management

## Abstract

Cervical subcutaneous emphysema and pneumomediastinum without pneumothorax are exceedingly rare complications following rhinoplasty, with limited cases reported in the literature. This report presents a case of revision septorhinoplasty using autologous costal cartilage, where the patient complained of a sore throat 36 hours postoperatively. On physical examination, cervical subcutaneous emphysema was palpated, and radiologic evaluation confirmed both cervical subcutaneous emphysema and pneumomediastinum. An upper aerodigestive tract injury was ruled out as a potential cause. The probable mechanism involved damage to the inner perichondrium of the costal cartilage and endothoracic fascia with an intact pleura, explained in light of current literature, radiographic imaging, and anatomical illustration.

This case report, combined with a literature review, provides insights into managing this unique complication and emphasizes the need for clinicians to recognize possible etiological factors related to surgical technique.

## Introduction

Surgical emphysema refers to the infiltration of air or gas into subcutaneous tissues, typically resulting from trauma to the esophageal or respiratory systems. When air is present within the mediastinum, it is defined as pneumomediastinum [1]. Cervical and facial subcutaneous emphysema are mainly caused by maxillofacial trauma, head and neck surgery, dental procedures, general anesthesia, vigorous coughing, or repetitive Valsalva maneuvers [2-4]. In the literature, cases of surgical emphysema and pneumomediastinum following septorhinoplasty are rare, with only limited reports available [5,6].

We present a case of a patient who developed cervical emphysema and pneumomediastinum following revision septorhinoplasty with costal cartilage graft, exploring possible etiological mechanisms in light of current literature.

## Case presentation

A 33-year-old male, dissatisfied with the aesthetic outcome of a previous septorhinoplasty performed two years prior, presented for revision surgery. Due to the lack of septal cartilage, revision septorhinoplasty with a costal cartilage graft was planned. Costal cartilage was harvested from the sixth rib, with care taken to preserve the posterior perichondrium. Intraoperative assessment for pneumothorax was conducted by filling the surgical field with saline solution and applying positive-pressure ventilation; no air bubbles were observed. Osteotomies were performed using osteotomes and a piezoelectric instrument, and silicone tampons were applied postoperatively. Initial postoperative chest X-rays showed no abnormalities.

**Figure 1:**
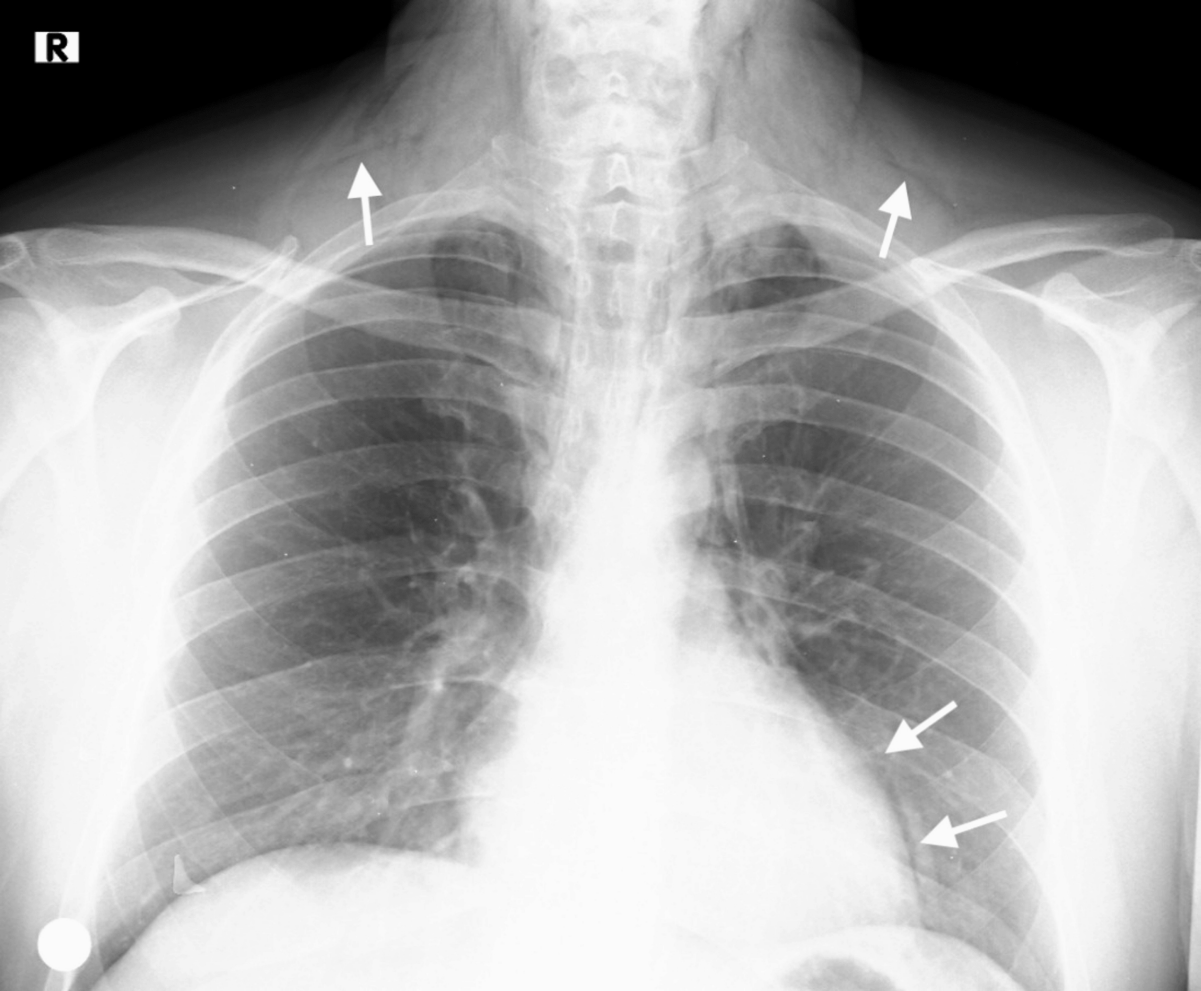
Postoperative thirty-sixth-hour chest X-ray Subcutaneous emphysema can be seen in the neck area and pneumomediastinum in the paracardiac region (white arrows).

At 36 hours postoperatively, the patient complained of a sore throat. Physical examination revealed palpable crepitations in the supraclavicular region. Subsequent chest X-ray showed evidence of cervical subcutaneous emphysema and pneumomediastinum (Figure 1).

**Figure 2:**
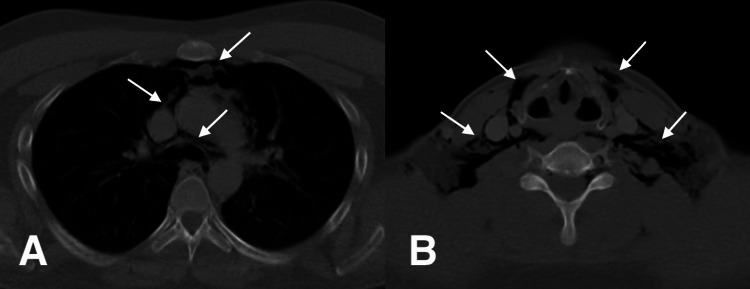
A: Postoperative thirty-sixth-hour, pneumomediastinum detected on thorax CT; B: Postoperative thirty-sixth-hour subcutaneous emphysema detected in the neck region (white arrows)

A thoracic surgery consultation was obtained, and a neck and thorax CT scan with both oral and intravenous contrast confirmed the presence of subcutaneous emphysema and pneumomediastinum without any signs of pneumothorax or aerodigestive injury (Figures 2A-2B). Due to the patient’s penicillin allergy, intravenous antibiotics were initiated with ciprofloxacin (400 mg twice daily) and metronidazole (100 mg three times daily), and oral intake was temporarily stopped. Laboratory analysis showed a white cell count of 12,600 (cells/mm³; reference range: 4490 to 12,680 cells/mm3) and a neutrophil count of 8,180 (cells/mm³; reference range: 2,100 to 8,890 cells/mm³), with all other hematologic and biochemical parameters within normal limits.

**Figure 3:**
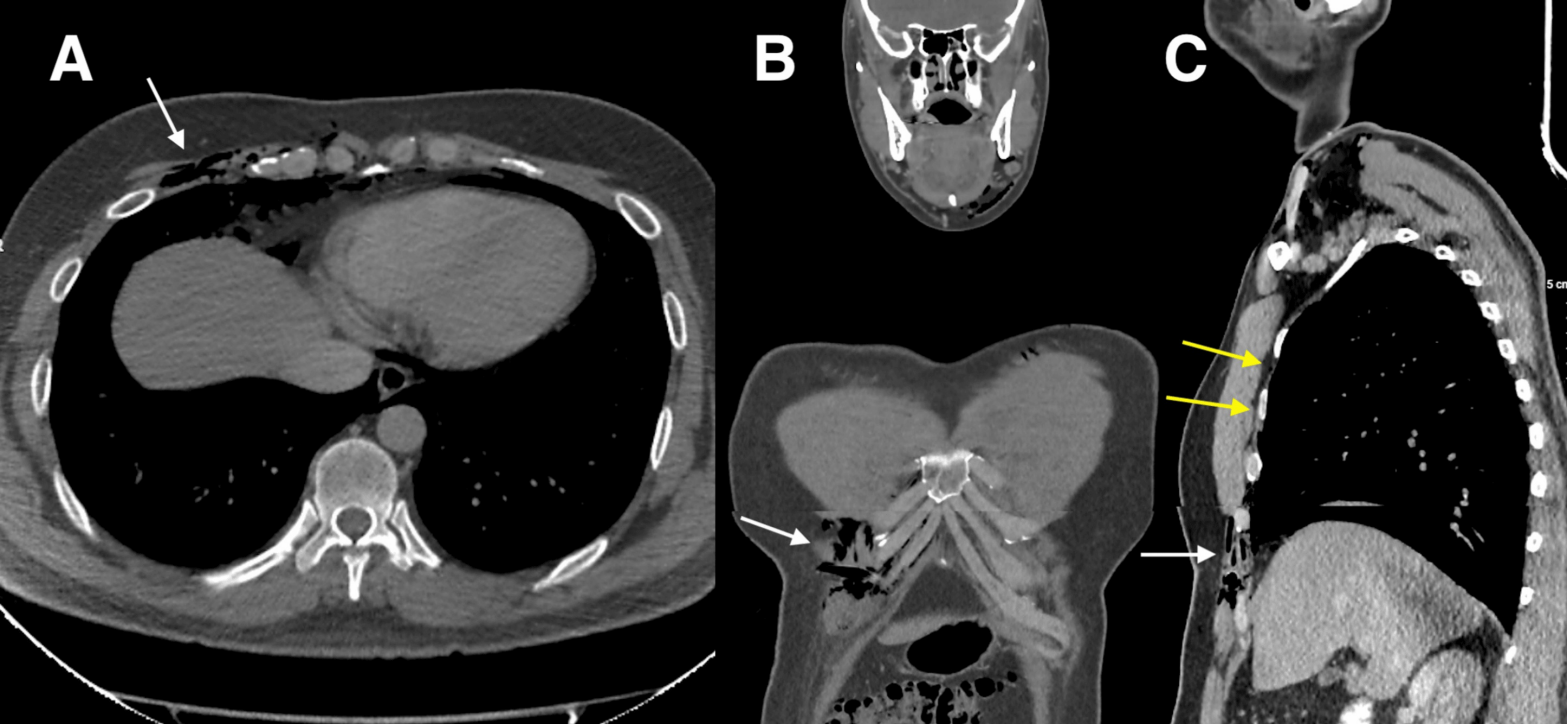
Possible pathway of air leakage to the mediastinum caused by a damaged endothoracic fascia (white arrows) and to cervical subcutaneous tissues via the subendothoracic space (yellow arrows)

Diagnostic flexible endoscopy and esophagoscopy were performed, which revealed no evidence of pharyngeal, hypopharyngeal, or esophageal injury. To clarify the etiology, the patient's CT scans were examined in detail by a radiologist. Thorax CT revealed unexpected air leakage to the mediastinum and cervical subcutaneous planes from the costal cartilage donor site (Figure 3). Since any signs of pneumothorax were not detected, it was thought that the endothoracic fascia was damaged and the parietal pleura was intact.

**Figure 4:**
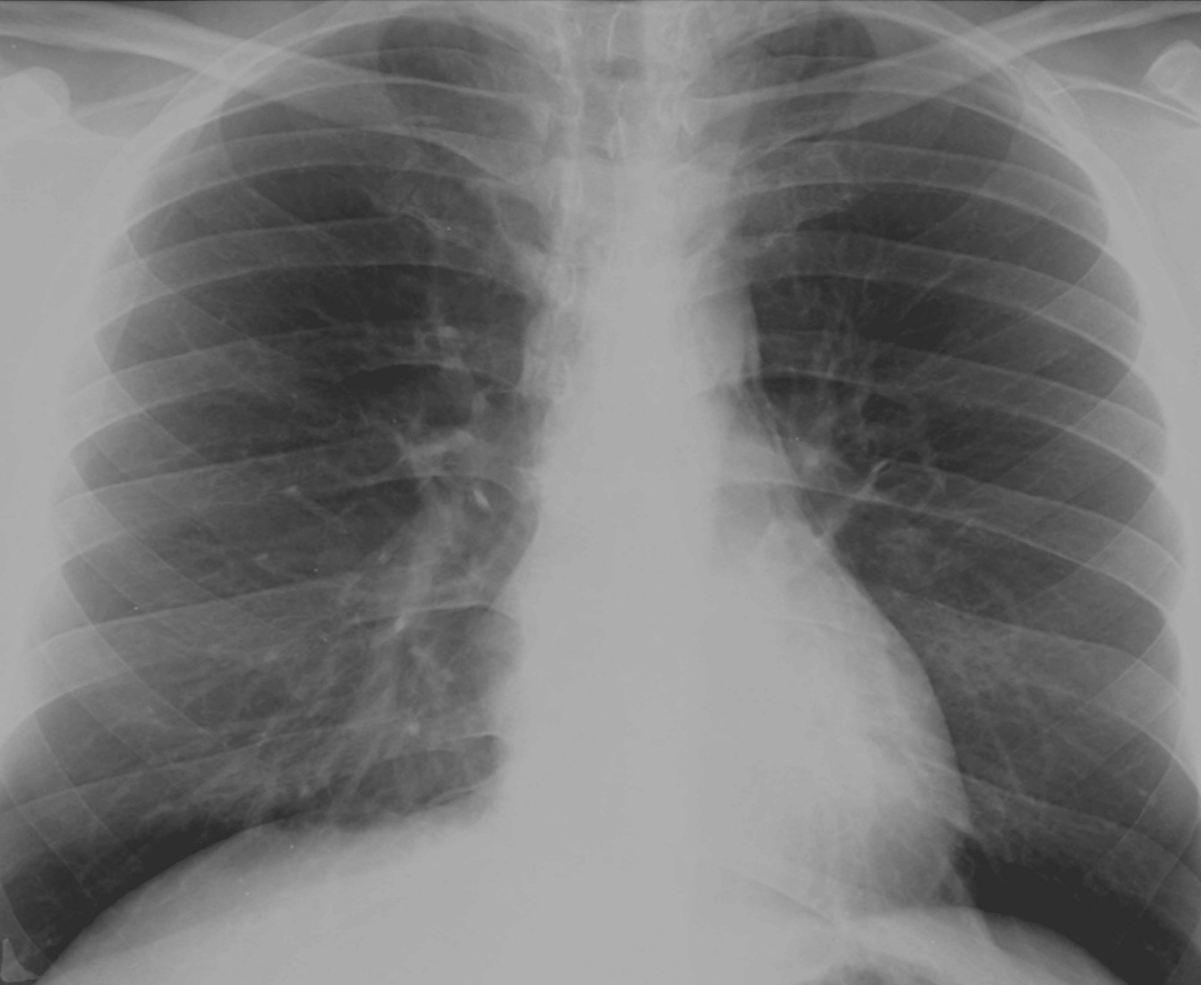
Postoperative fifth day complete regression of pneumomediastinum seen on chest X-ray

The patient was closely monitored with daily chest X-rays. Over the course of the next three days, the patient’s vital signs and respiratory status remained stable, with gradual resolution of emphysema and pneumomediastinum (Figure 4).

Oral intake was resumed with soft foods on postoperative day three. By day five, physical examination revealed no remaining crepitations in the neck region, and chest X-rays confirmed the complete resolution of pneumomediastinum. The patient was discharged on the fifth postoperative day with instructions for oral antibiotics and dietary recommendations. A follow-up examination conducted one-week post-discharge showed no abnormal findings.

## Discussion

The use of autologous costal cartilage in rhinoplasty has increased significantly in recent years due to the rising demand for revision procedures, as well as its use in primary cases where septal cartilage is insufficient [7]. Revision septorhinoplasty, while effective, can be associated with various complications, including epistaxis, septal hematoma, orbital hemorrhage, enophthalmos, and several complications specific to autologous rib cartilage, such as graft warping, migration, dorsal irregularities, infection, and pneumothorax, at the donor site. Among these, pneumothorax is rare, with a reported incidence of approximately 0.1% [8].

Cervical and facial subcutaneous emphysema and pneumomediastinum are more commonly associated with maxillofacial trauma, head and neck surgeries, dental extractions, general anesthesia, and forceful or repetitive actions such as coughing or performing the Valsalva maneuver [5]. When the air becomes trapped within the cervicofacial soft tissues, it may descend into the mediastinum, facilitated by fascial planes that provide the path of least resistance. This dissemination is possible through anatomical connections among the parapharyngeal, carotid sheath, and retropharyngeal spaces, creating a continuous air pathway that can lead to pneumomediastinum [9,10]. However, in our case, a different mechanism involving the costal cartilage harvesting is considered the underlying cause and will be mentioned in detail.

In the literature, pneumomediastinum and subcutaneous emphysema following septorhinoplasty are exceptionally rare, with only a few cases reported. For instance, Celebioğlu et al. documented subcutaneous emphysema in a 24-year-old male four hours after rhinoplasty, attributing it to air pumped through lateral osteotomy incisions, which acted as one-way valves [11]. Similarly, Kim et al. described pneumomediastinum in a 35-year-old male following septorhinoplasty, with symptoms resolving after antibiotic administration and bed rest [5]. Balıkçı et al. reported subcutaneous emphysema after septoplasty, likely resulting from over-elevation of the mucoperiosteum, allowing air entry through a one-way valve effect [6]. In another case, Rashid et al. described cervicofacial emphysema and pneumomediastinum following a nasal fracture in a previously healthy individual [12].

In the present case, an alternative mechanism associated with costal cartilage harvesting is suggested. Following cartilage harvest with intact posterior perichondrium, routine positive-pressure testing for pneumothorax in the saline-filled surgical field showed no air leakage. Nevertheless, minor trauma may have damaged the posterior perichondrium and endothoracic fascia, resulting in cervical emphysema without any harm to the parietal pleura.

**Figure 5:**
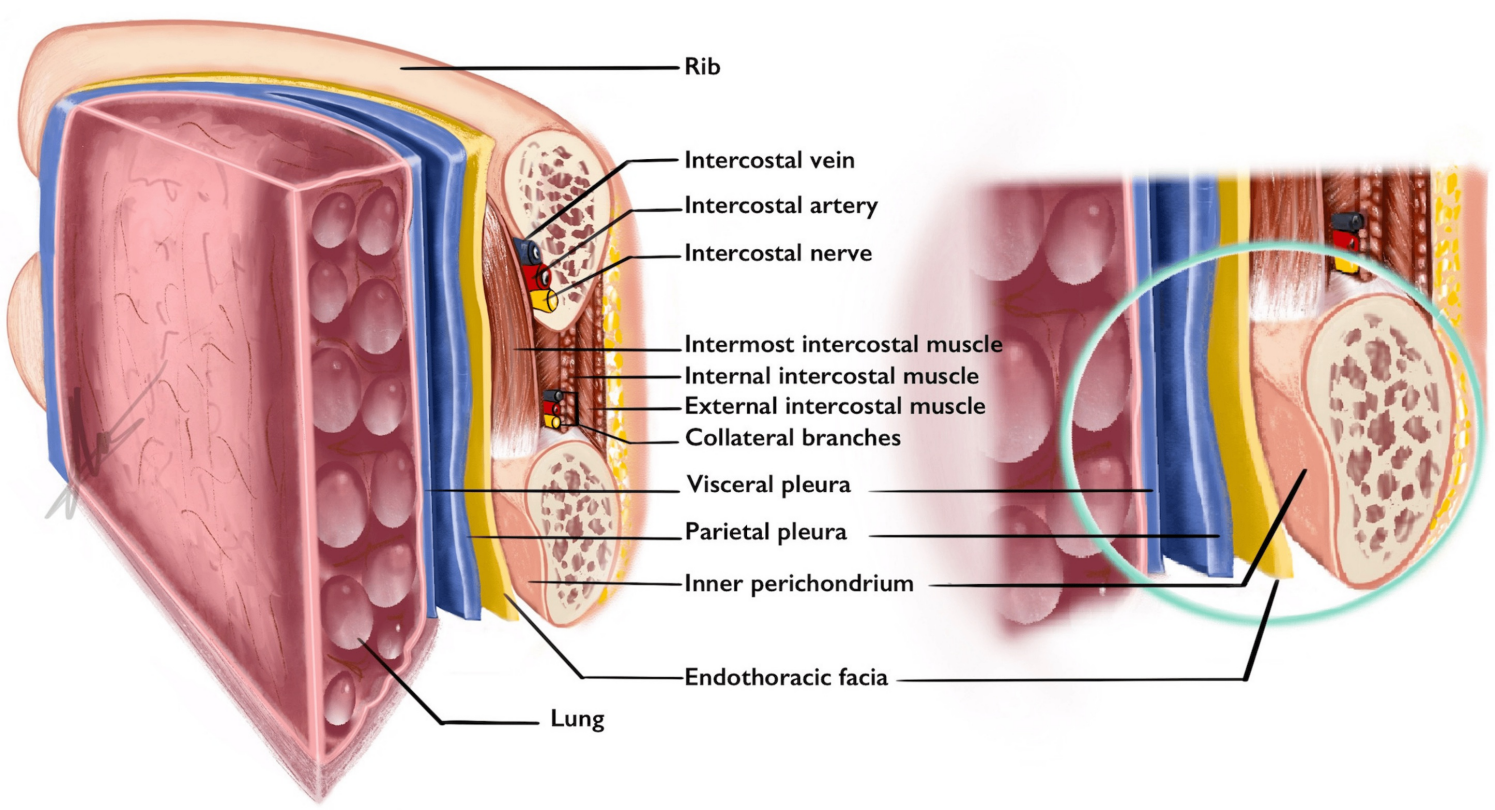
The inner perichondrium of the rib is continuous with the endothoracic fascia, which is indistinguishable from the parietal pleura during surgical dissection Image credit: Emre Demirel

The endothoracic fascia, a fibroelastic structure forming the deep fascia of the thoracic cavity, lines the internal surfaces of the intercostal muscles, ribs, and diaphragm (Figure 5). Electron microscopy has shown the fascia thickness ranges between 15 and 27 μm, making it indistinguishable from the parietal pleura during surgical procedures [13,14].

Superiorly, the endothoracic fascia is continuous with the suprapleural membrane, or Sibson’s fascia, which is attached to the inner border of the first rib, anterior costal cartilage, C7 transverse process, and mediastinal pleura. This anatomical continuity may allow air to travel into the cervical subcutaneous planes when the endothoracic fascia is damaged but the parietal pleura remains intact (Figure 3) [15].

In this case, it is hypothesized that negative pressure within the mediastinum during expiration enabled air entry into the cervical subcutaneous spaces. Despite this, the intact parietal pleura likely prevented air bubbles in the surgical field and ruled out pneumothorax on examination and imaging. Initial symptoms of sore throat and crepitus in the supraclavicular area on the first postoperative day prompted further imaging, which confirmed the presence of cervical subcutaneous emphysema and pneumomediastinum. Conservative management including cessation of oral intake, intravenous antibiotics, and close monitoring was implemented following the exclusion of aerodigestive injury via endoscopic evaluation. By postoperative day three, marked improvement was observed, and oral intake was cautiously resumed. Full resolution of symptoms and imaging findings by day five enabled safe patient discharge.

## Conclusions

This case highlights the rare complication of pneumomediastinum following revision septorhinoplasty with a costal cartilage graft. In postoperative evaluations, even minor complaints, such as sore throat, warrant detailed assessment due to the potential for this rare complication. It is crucial to conduct daily monitoring of vital signs and lung imaging, administer intravenous antibiotics, and withhold oral intake until the underlying cause is identified. Clinicians should also be aware of potentially life-threatening complications arising from pneumomediastinum.
